# ASSOCIATION BETWEEN SELF-REALIZATION AND HEALTH OUTCOMES IN FAMILY CAREGIVING DYADS

**DOI:** 10.1093/geroni/igae098.2688

**Published:** 2024-12-31

**Authors:** Cristina de Rosa, Weijun Wang, Yu-Ping Chang

**Affiliations:** University at Buffalo, SUNY, Buffalo, New York, United States; University at Buffalo, SUNY, Buffalo, New York, United States; University at Buffalo, SUNY, Buffalo, New York, United States

## Abstract

Self-realization, such as perceptions of life having meaning and purpose, can potentially influence health in older adults and their family caregivers, but how dyad members may contribute to each other’s outcomes is less well understood. This analysis examined whether self-realization in older adults and their family caregivers was associated with their own, and each other’s, mental health and perceived general health. Data for adults aged 65 and older were obtained from the National Health and Aging Trends Study (NHATS) (2022) linked with their respective family caregivers from the National Study of Caregiving (NSOC) IV (2022). A total of 1,571 dyads consisted of one older adult and their primary caregiver, defined as the family member or friend who provided the greatest number of hours of care. Actor-partner interdependence models (APIM) were used to examine associations between self-realization and mental health and perceived general health, controlling for dyad members’ own gender, age, and race/ethnicity, and dementia status of the care recipient. Results showed that self-realization significantly predicted caregivers’ and older adults’ own mental health and perceived general health. Furthermore, older adults’ self-realization significantly predicted their caregivers’ perceived general health (b = 0.043, S.E. = 0.014, 95% CI = [0.016, 0.070], p =.002). Findings suggest mental health and perceived general health are impacted by a person’s own self-realization even when accounting for partner effects. Caregivers are particularly influenced by older adults’ self-realization. Strategies supporting both dyad members may provide benefits for both older adults and their family caregivers.

